# Effects of a valgus unloader brace in the medial meniscectomized knee joint: a biomechanical study

**DOI:** 10.1186/s13018-019-1085-1

**Published:** 2019-02-12

**Authors:** Duraisamy Shriram, Go Yamako, Etsuo Chosa, Yee Han Dave Lee, Karupppasamy Subburaj

**Affiliations:** 1 Engineering Product Development (EPD) Pillar, Singapore University of Technology and Design (SUTD), 8 Somapah Road, Singapore, 487372 Singapore; 2 Department of Mechanical Design Systems, Faculty of Engineering, University of Miyazaki, 1-1 Gakuen Kibana-dai-nishi, Miyazaki, 889-2192 Japan; 3 Department of Orthopaedic Surgery, Faculty of Medicine, University of Miyazaki, 5200 Kihara, Kiyotake, Miyazaki, 889-1692 Japan; 4 Department of Orthopaedic Surgery, Changi General Hospital, 2 Simei Street 3, Singapore, 529889 Singapore

**Keywords:** MRI, Gait analysis, Finite element analysis, Meniscectomy, Osteoarthritis, Arthroscopy, Unloader brace

## Abstract

**Background:**

Patients undergoing total or partial arthroscopic meniscectomy for treating traumatic meniscal tears are at greater risk of developing knee osteoarthritis (OA) due to increased mechanical load. The purpose of this study was to evaluate the effects of a valgus unloader brace in the medial meniscectomized knee joint during the gait cycle.

**Methods:**

A three-dimensional finite element model of the knee joint was developed using the substructures segmented from magnetic resonance images. Experimentally measured forces and moments for one complete gait cycle, without brace and with brace at three different alignment angles (0°, 4°, and 8°), were applied to the finite element model, and the changes in the tibiofemoral contact mechanics were estimated.

**Results:**

The brace in 0°/4°/8° valgus alignment modes reduced the total contact force in the medial compartment by 16%/46%/82% at opposite toe off and 18%/17%/29% at opposite initial contact events, while it increased the total contact force in the lateral compartment by 31%/81%/110% at opposite toe off and 30%/38%/45% at opposite initial contact events, respectively, when compared to the unbraced meniscectomized knee.

**Conclusions:**

Increasing the valgus alignment from 0° to 4° and 8° resulted in a greater reduction of contact conditions (total contact force, total contact area, peak contact pressure) in the medial compartment and vice versa in the lateral compartment. This decrease in contact conditions in the medial compartment infers enhanced knee joint function due to a valgus unloader brace, which translates to increased knee-related confidence. Results suggest choosing a higher valgus alignment angle could potentially increase the risk for the onset of osteoarthritis in the lateral compartment, and this computational model could be used in validating the effectiveness of braces on joint health.

**Electronic supplementary material:**

The online version of this article (10.1186/s13018-019-1085-1) contains supplementary material, which is available to authorized users.

## Introduction

Traumatic meniscal injuries are common in young athletes, especially those who are associated with contact sports that entail frequent pivoting [1]. The treatment options vary depending on the anatomical location and extent of the injury [1]. Typical surgical procedure to treat the meniscal injury is arthroscopic meniscectomy. Arthroscopic total or partial meniscectomy is one of the primary risk factors for new onset and/or progression of knee osteoarthritis (OA) [2]. Even though the benefits of arthroscopic meniscectomy are debatable [3], a large number of these procedures are performed to treat the patients with a symptomatic meniscal tear [4]. A recent study showed a three-fold increase in peak contact pressure in the meniscectomized knee joint during a short-term gait load at full extension when compared to the intact joint [5]. Thus, evaluating the treatment alternatives to avert or delay the onset and progression of knee OA due to arthroscopic meniscectomy is vindicated.

Knee joints of patients who have undergone total or partial medial meniscectomy experience higher mechanical loads in the medial compartment when compared to the healthy contralateral ones due to increased knee adduction moment [6]. Resection of medial meniscus entirely from the joint causes a significant increase in the varus alignment angle [7] which translates to higher knee adduction moment (a surrogate biomarker for higher medial joint load) [6]. In literature, the association between higher mechanical loading and the onset and progression of knee OA has been affirmed by numerous studies [6–9]. The severity of medial knee OA has been found to be in correlation with the total contact force and the peak contact pressure acting within the medial compartment [8, 9]. Among non-surgical interventions to avert and reduce progression rate of early-stage OA, the most widely used mechanical intervention-based treatment, a valgus knee brace, applies an external counteracting abduction moment about the joint in order to unload the medial compartment [9].

A valgus unloader brace, which aims to unload the medial compartment, might be effective in delaying the onset and progression of medial knee OA over time. Significant improvement in joint function such as decreased pain, reduced joint stiffness, and increased confidence in knee-related physical activities has been reported after using different valgus unloader braces [8–10]. Nonetheless, little information is available on the underlying mechanisms causing these symptomatic reliefs. In literature, many studies that used pain reduction as the success criterion for the use of a valgus unloader brace did not attempt to quantify the contact conditions in the affected compartment induced by a valgus unloader brace [11]. A biomechanical analysis of the contact conditions in both the affected and the healthy contralateral compartments of the knee joint would provide meaningful insight about the possible underlying mechanisms responsible for the torment relief and enhanced knee joint function reported in other studies. Specifically, effects of a valgus unloader brace at different valgus alignment angles on the (1) contact conditions in the affected (medial) compartment, (2) contact conditions in the healthy contralateral (lateral) compartment, and (3) tibial kinematics relative to the femur for a complete gait cycle are not investigated in detail yet.

The main aim of this study was to test the theory that wearing a valgus brace would significantly reduce the medial joint load in the medial meniscectomized knee joint during the gait cycle. The secondary exploratory aim of this study was to assess the effect of a valgus unloader brace on the total contact force, total contact area, and the peak contact pressure in the medial and the lateral compartments and the tibial kinematics relative to the femur for the following cases: (1) without a valgus knee brace; (2) valgus knee brace at 0° alignment; (3) valgus knee brace at 4° alignment; and (4) valgus knee brace at 8° alignment. To achieve these specific aims, we conducted finite element (FE) simulations on a medial meniscectomized knee joint model by applying the gait cycle data (forces and moments) for the aforementioned cases. The presented numerical approach provides a novel procedure for patient-specific analysis of the knee joint to test the effectiveness of various mechanical interventions available in the market for delaying or averting the onset of early-stage OA.

## Materials and methods

### Methodology

**Fig. 1:**
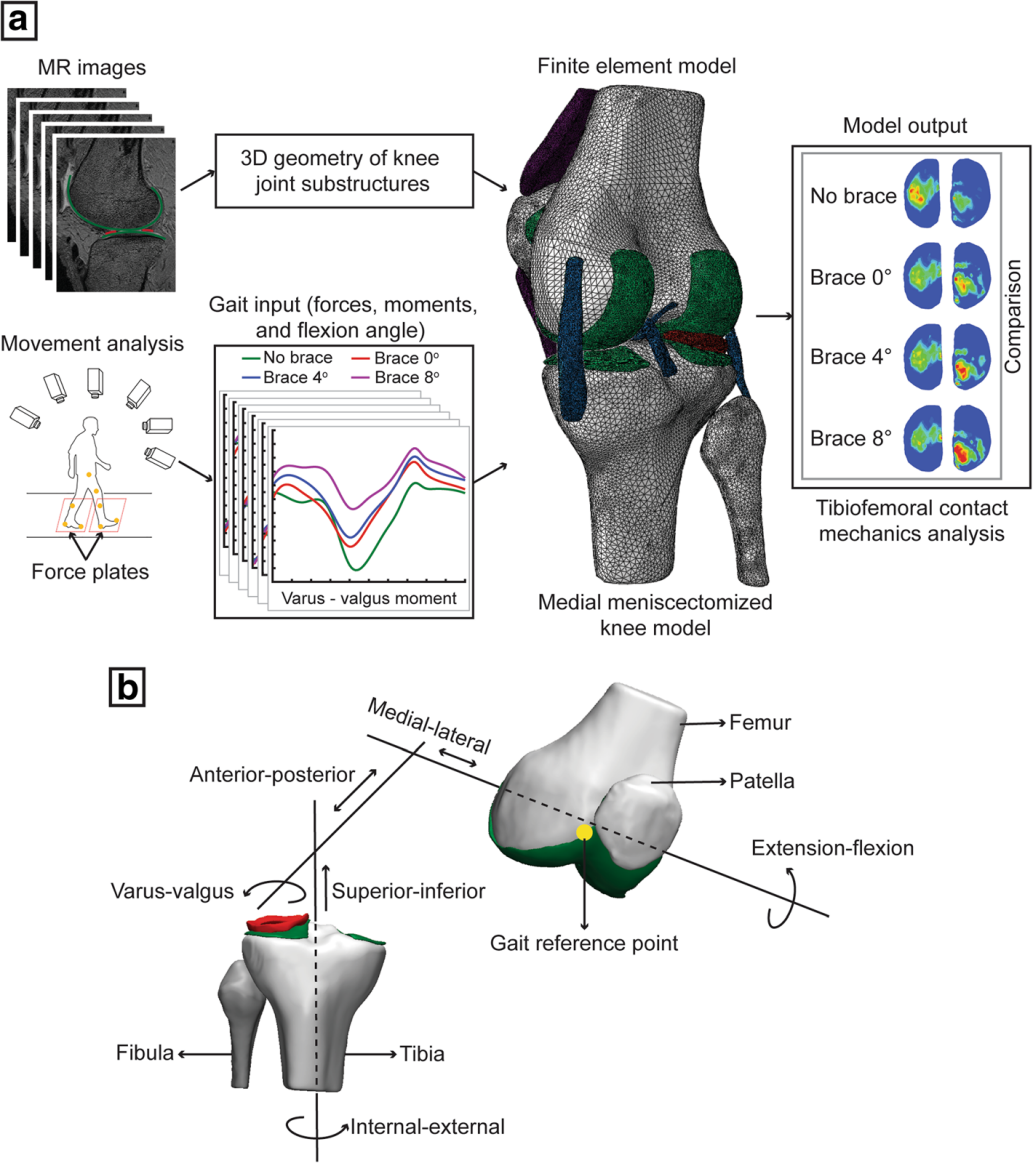
**a** Study workflow and **b** translational and rotational degrees of freedom of the human knee joint

All methods were carried out in accordance with relevant guidelines and regulations. All experimental protocols were approved by a named institutional/licensing committee. The overall workflow of the study is schematically illustrated in Fig. 1a. This study combined experimental data and FE model to investigate the biomechanical effects of a valgus unloader brace in the medial meniscectomized knee joint. Briefly, three-dimensional (3D) reconstructed geometries of joint substructures, segmented from magnetic resonance (MR) medical images, were used to construct the FE model of the knee joint. Experimentally measured forces and moments for one complete gait cycle, without brace and with brace at three different alignment angles (0°, 4°, and 8°), were applied as a loading condition into the FE model of the knee joint at the gait reference point (Fig. 1b), and the simulations were conducted. The contact conditions including the total contact force, total contact area, and the peak contact pressure in both the medial and the lateral compartments and the tibial kinematics relative to the femur were estimated and compared for each case considered.

### Finite element model of the knee joint

A healthy volunteer (gender male, age 29 years) with a body mass index (BMI) of 23 kg/m^2^ and with no knee stiffness, no knee torment, no prior injury to the knee, no knee joint disorder, and no past surgical history that affects the soft tissues of the knee joint was recruited for this study. MR imaging of the left knee was acquired with a 3.0 Tesla MR scanner (Signa® HDx, GE Healthcare, Waukesha, WI, USA) using a fast spin-echo (FSE) sequence with the accompanying parameters as follows: repetition time (TR)/echo time (TE) = 15/6.7 ms, echo train length (ETL) = 7, flip angle = 18°, slice thickness = 1 mm, receiver bandwidth = 31.25 kHz, number of excitations (NEX) = 1, 512 × 512 acquisition matrix size, and field of view (FOV) = 14 cm (Fig. 1a). This study was endorsed by the local Institutional Review Board of the Singapore University of Technology and Design, Singapore, and informed consent was acquired from the volunteer.

The 3D image processing software, Mimics (Materialise NV, Leuven, Belgium), was used to segment the bones (femur, fibula, patella, and tibia), menisci (lateral meniscus and medial meniscus), articular cartilages (femoral cartilage, patellar cartilage, and tibial cartilage), ligaments (anterior cruciate ligament (ACL), posterior cruciate ligament (PCL), medial collateral ligament (MCL), and lateral collateral ligament (LCL)), and tendons (patellar tendon (PT) and quadriceps tendon (QT)). Each substructure is segmented twice by two different individuals to avoid variations and to ensure the segmented geometry is anatomically accurate. The incorporation of adjacent joint substructures in any computer-aided modeling tool ineluctably results in boundary gaps and/or overlaps at contact surfaces. To surmount this, common contact surfaces were created between adjacent joint substructures with the aid of “non-manifold assembly” intersection algorithm available in Mimics. The 3-matic module available in the Mimics software was used to create surfaces on bony substructures to define the attachment sites for ligaments and tendons. Iterative smoothing and re-meshing operations were performed on the final surface mesh using the 3-matic module to minimize rough surfaces, number of minuscule elements, and superfluous computational cost.

Reconstructed 3D surface geometry of the knee joint substructures was imported into SolidWorks (SolidWorks Corp., Concord, MA, USA), where the solid geometry of the joint substructures was created to develop a 3D solid assembly model of the knee joint. The 3D solid geometry was then imported into Abaqus (Dassault Systèmes Simulia Corp., Providence, RI, USA) to develop a finite element model of the knee joint (Fig. 1a). The contact surfaces of cartilage-cartilage, cartilage-meniscus, and meniscus-cartilage were modeled using frictionless sliding contact elements. Bones were meshed using 3-noded discrete rigid triangular elements (defined as R3D3 in Abaqus), and the soft tissues (menisci, cartilages, ligaments, and tendons) were meshed using 10-noded quadratic tetrahedron elements (defined as C3D10 in Abaqus). A mesh sensitivity study was conducted on the mesh element size for the different anatomical geometries to ascertain that the mesh density is adequate and the predicted results are not affected by the chosen element size. A mesh element size of 1 mm for cartilages, menisci, and tendons and 0.5 mm for ligaments was chosen for the final knee joint FE model based on the mesh sensitivity study. After the mesh sensitivity study, the medial meniscus was excluded from the final model to conduct simulations to study the biomechanical effects of a valgus unloader brace in the medial meniscectomized knee joint.

### Material properties

Articular cartilages were modeled as non-linear, isotropic, and hyperelastic neo-Hookean material with the strain energy density function:

1 $$ W={C}_{10}\left({\overline{I}}_1-3\right)+\frac{1}{D_1}{\left({J}_{\mathrm{el}}-1\right)}^2 $$

where *C*_10_ denotes the neo-Hookean material constant associated with the modulus of rigidity $\mu\ \left({C}_{10}=\frac{\mu }{2}\right)$, *D*_1_ denotes the inverse of volumetric elasticity $\kappa\ \left({D}_1=\frac{2}{\kappa}\right)$, ${\overline{I}}_1$ denotes the first deviatoric strain invariant, and *J*_el_ denotes the total elastic volume ratio. The values of neo-Hookean coefficients, *C*_10_ and *D*_1_ (*C*_10_ = 0.86 *MPa*; *D*_1_ = 0.048 *MPa*^−1^), used in this study for modeling the articular cartilages were based on experimental compressive modulus tests [12].

**Fig. 2:**
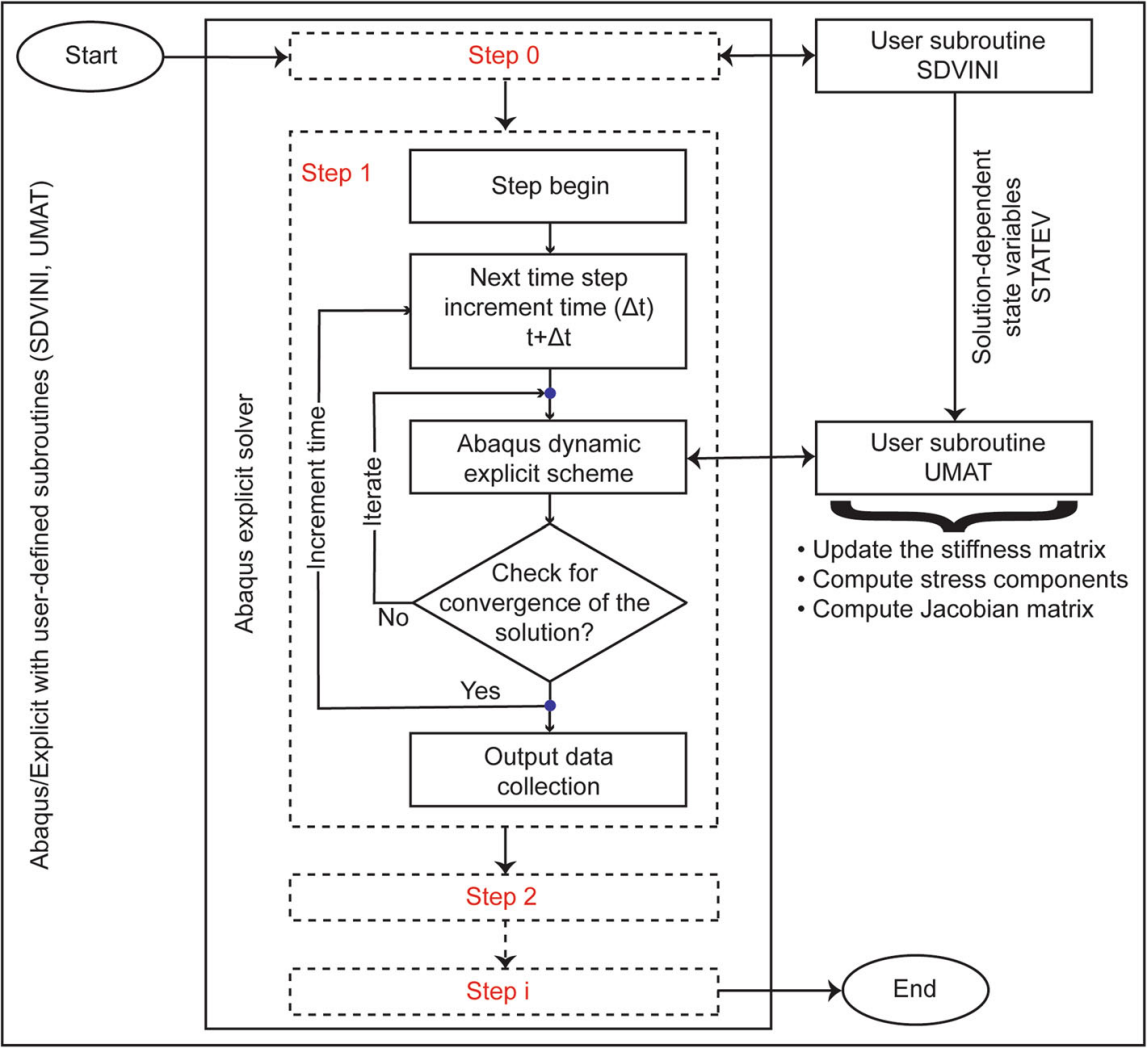
Schematic flowchart of multistep finite element analysis using Abaqus explicit solver in which the user-defined UMAT and SDVINI subroutines are linked

Nearly incompressible, transversely isotropic and hyperelastic neo-Hookean material, implemented in Abaqus FE tool as a user-defined material using the UMAT and SDVINI subroutines, was used to model the ligaments, tendons, and the menisci [13]. The subroutines coded in FORTRAN were compiled and linked with the Abaqus explicit solver (Fig. 2). The SDVINI subroutine was mainly used to define the initial values of state variables (STATEV). The strain energy density function

2 $$ \mathrm{W}={C}_{10}\left({\overline{I}}_1-3\right)+\frac{1}{D_1}{\left({J}_{\mathrm{el}}-1\right)}^2+Q\left(\lambda \right) $$

consists of neo-Hookean terms (*C*_10_ and *D*_1_) which represent the non-collagenous matrix substance, and the fiber family strain energy term (*Q*(*λ*)) which represents the stiffness of the collagen fibers. The function *Q*(*λ*) satisfies the conditions:

3 $$ \lambda \frac{d Q}{d\lambda}=\left\{\begin{array}{ll}0,& \lambda \le 1\\ {}{C}_3\left({e}^{C_4\left(\lambda -1\right)}-1\right),& 1<\lambda <{\lambda}^{\ast}\\ {}{C}_5\lambda +{C}_6,& \lambda \ge {\lambda}^{\ast}\end{array}\right. $$

where *λ* denotes the fiber stretch, *λ*^∗^ denotes the maximum stretch value beyond which the fibers straighten, and *C*_3_, *C*_4_, *C*_5_, and *C*_6_ denote the material coefficients. The fiber stretch can be computed from the orientation of fibers, ***a***(***x***) (current or deformed configuration) and ***a***_**0**_***(X)*** (initial or reference configuration), and the deformation gradient ***F*** using the relation (*λ*. ***a(x)*** = ***F***. ***a***_**0**_***(X))***. In all the cruciate and the collateral ligaments, the collagen fibers were oriented along the principal axis of the ligament geometry. The collagen fibers in the menisci were oriented along the circumferential direction in order to resist circumferential stresses when subjected to loading [14, 15]. The collagen fibers do not support compressive stresses when these fibers were subjected to a compressive force (*λ* ≤ 1). The stiffness of the collagen fibers increases exponentially if *λ* is greater than 1 and less than the maximum stretch value. The stiffness of the collagen fibers increases linearly if *λ* exceeds the maximum stretch value. The material constants *C*_3_, *C*_4_, and *C*_5_ represent the exponential growth rate of collagen fiber stiffness, uncrimping rate of collagen fibers, and the modulus of elasticity of straightened fibers. The material constant *C*_6_ denotes the continuation of stress at the maximum stretch value and can be computed using the following relation:

4 $$ {C}_6={C}_3\left({e}^{C_4\left({\lambda}^{\ast }-1\right)}-1\right)-{C}_5{\lambda}^{\ast } $$

The material constants *C*_10_, *C*_3_, *C*_4_, *C*_5_, and *D*_1_ derived through curve fitting of stress-strain tensile experimental data [16–18] are presented in Table 1.

**Table 1:** Material parameters and their values for the transversely isotropic and hyperelastic neo-Hookean material model of the ligaments (*ACL* anterior cruciate ligament, *PCL* posterior cruciate ligament, *MCL* medial collateral ligament, and *LCL* lateral collateral ligament), the menisci, and the tendons (*PT* patellar tendon and *QT* quadriceps tendon) [16–18]

Soft tissue	Material parameters					
	*C*_10_ (MPa)	*D*_1_ (MPa^−1^)	*C*_3_ (MPa)	*C*_4_ (−)	*C*_5_ (MPa)	λ^*^ (−)
ACL	1.95	0.00683	0.0139	116.22	535.039	1.046
PCL	3.25	0.0041	0.1196	87.178	431.063	1.035
MCL	1.44	0.00126	0.57	48.0	467.1	1.063
LCL	1.44	0.00126	0.57	48.0	467.1	1.063
Menisci	4.61	0.01085	0.1197	150.0	400.0	1.019
PT	2.75	0.00484	0.065	115.89	777.56	1.042
QT	2.75	0.00484	0.065	115.89	777.56	1.042

The bones were modeled as rigid bodies because the strength and stiffness of the bones are several orders of magnitude higher than that of the soft tissues [5, 18]. The meniscal horn attachments were modeled as non-linear springs (four per horn) with zero compression and with a stiffness of *k* = 400 N/mm [19, 20]. The anterior and posterior meniscofemoral ligaments were modeled using one non-linear spring with no compression (*k* = 49 N/mm) [21]. The transverse meniscomeniscal ligament was modeled using three no-compression non-linear springs with a stiffness of *k* = 400 N/mm [19, 20]. The medial and lateral patellofemoral ligaments were modeled using two non-linear springs (*k* = 10 *N*/*mm*) with no compression [22, 23]. The anterolateral ligament (ALL) was modeled using one non-linear spring (no compression) with a of stiffness *k* = 42 N/mm [24, 25]. The capsular ligaments of the knee including the medial capsular ligament (MCap), lateral capsular ligament (LCap), oblique popliteal ligament (OPL), and the arcuate popliteal ligament (APL) were modeled using one no-compression non-linear spring with a stiffness of *k*_MCap_ = 15 N/mm, *k*_LCap_ = 14 N/mm, *k*_OPL_ = 28 N/mm, and *k*_APL_ = 34 N/mm, respectively [24–27].

### Gait analysis and boundary and loading conditions

The same subject, who participated in the MR image acquisition, also volunteered for the gait analysis study. The subject walked without a valgus knee brace on a force-plated treadmill at a self-selected speed of approximately 4 km/hr. Two force platforms at 1080 Hz (AMTI, Newton, MA, USA) and a 12-camera three-dimensional motion analysis system at 120 Hz (Vicon MX, Oxford Metrics, Oxford, UK) were used to collect the position and orientation of the 42 markers and the ground reaction force (GRF) data simultaneously. These data were introduced into LifeMod (LifeModeler Inc., San Clemente, Califonia), a plug-in of ADAMS software (MSC Software Corporation, Newport Beach, California), to construct the subject-specific multibody musculoskeletal model [28–30]. Forces (superior-inferior, anterior-posterior, and medial-lateral), moments (varus-valgus and internal-external), and corresponding extension-flexion rotation for one complete gait cycle were calculated through the inverse dynamics and forward dynamics approach (Fig. 1a) [29]. This gait analysis study was repeated with a valgus knee brace (Orthomen Inc., Foothill Ranch, CA, USA) at three different alignment angles (0°, 4°, and 8°). The quadriceps forces (superior-inferior and anterior-posterior), without brace and with brace at three different alignment angles (0°, 4°, and 8°), were estimated using an inverse dynamics model [31] for one complete gait cycle (refer to Additional file 1: Figure S1a–b).

The bottom nodes of the distal tibia were fixed and had no degrees of freedom. All degrees of freedom of the proximal femur except extension-flexion rotation were not constrained. The final gait data input (average of seven gait trials) for the FE model included forces and moments (except extension-flexion) and quadriceps forces for one complete gait cycle (refer to Additional file 1: Figure S2a–f). The extension-flexion rotation of the knee joint was applied as a boundary condition to the FE model. The moments (varus-valgus and internal-external) estimated from the gait analysis were scaled to 50% to account the effect of muscles and other connective tissues [32]. A pre-strain of 5% was applied to the ligaments (ACL, PCL, LCL, and MCL) and tendons (PT and QT), assuming they were in tension while acquiring MR images [33]. Gait forces and moments were applied to the FE model of the knee joint at the gait reference point (Fig. 1b), which is located in the middle of the femoral epicondyles. The quadriceps forces were implemented at the reference point located on the quadriceps tendon, which is coupled to the femur [32].

## Results

### Contact mechanics in the medial compartment

**Fig. 3:**
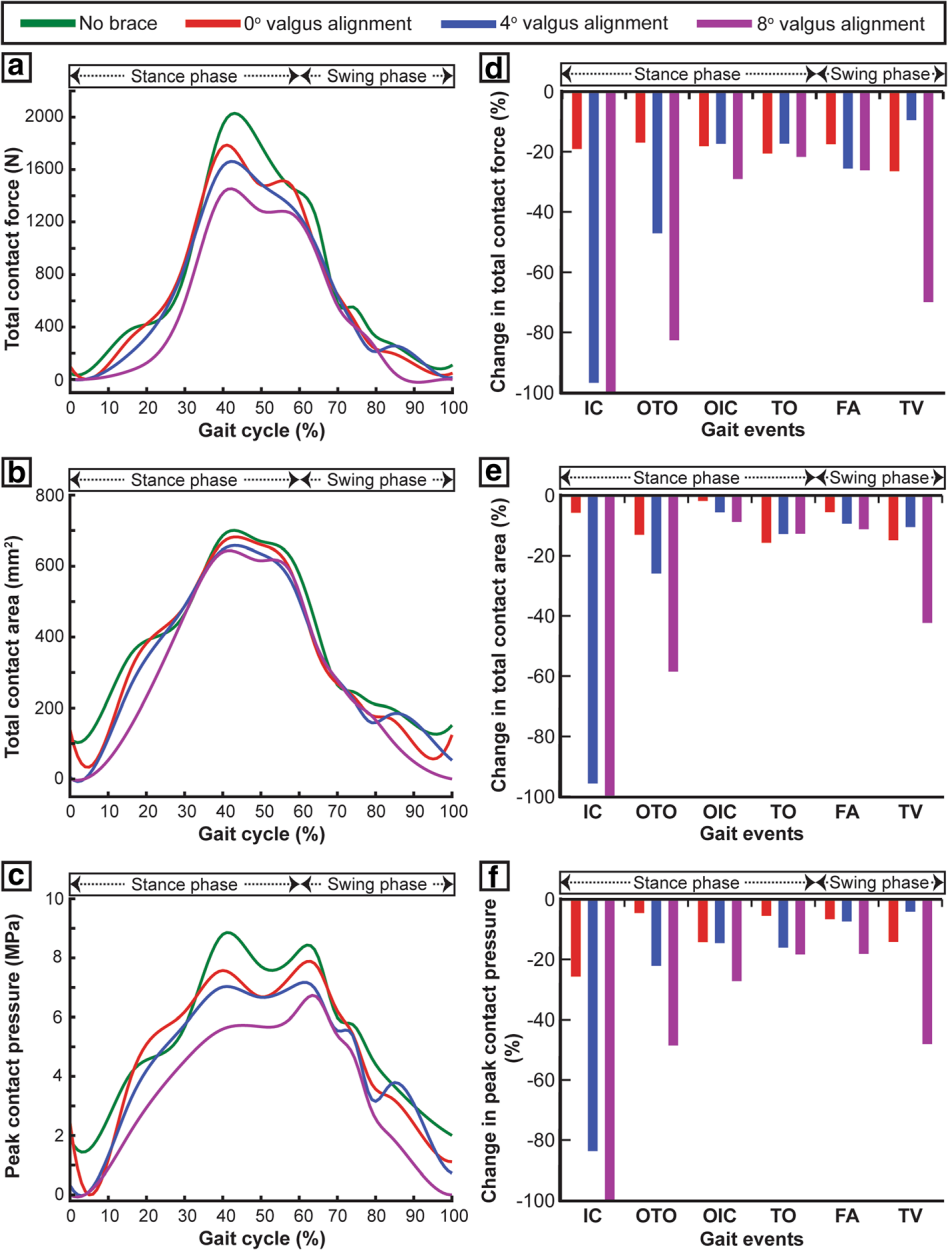
**a** Total contact force, **b** total contact area, and **c** peak contact pressure induced on the medial tibial cartilage during one complete gait cycle for unbraced and braced modes. **d**–**f** Percentage change in the medial compartment contact mechanics (total contact force, total contact area, and peak contact pressure) for different braced modes during the main events of the gait cycle (IC, initial contact; OTO, opposite toe off; OIC, opposite initial contact; TO, toe off; FA, feet adjacent; TV, tibia vertical) when compared with the unbraced mode

**Fig. 4:**
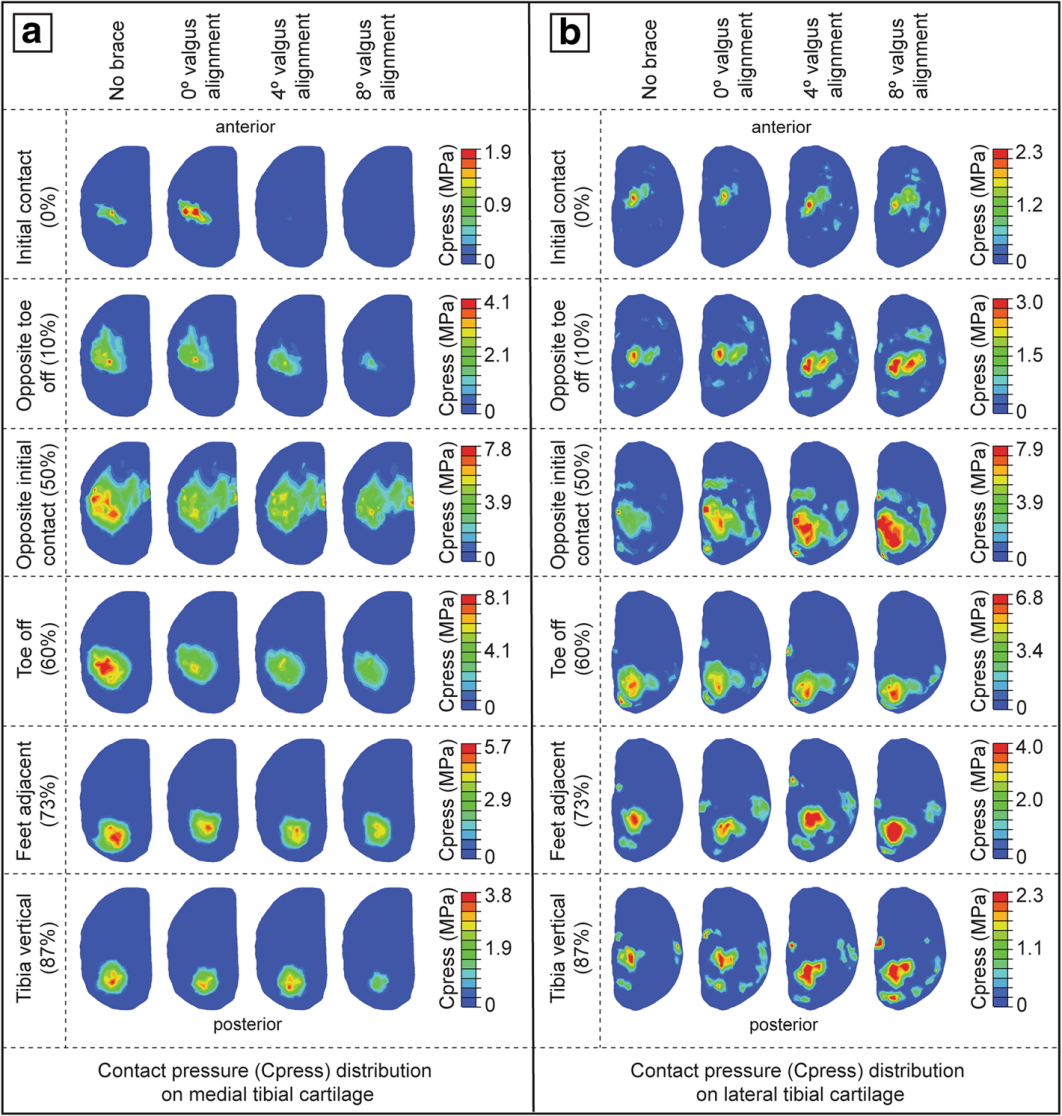
Contact pressure distribution on **a** the medial tibial cartilage and **b** the lateral tibial cartilage for unbraced and braced modes during the main events of the gait cycle. The peak contact pressure induced on the respective tibial cartilage for unbraced mode was set as the upper scale value for each braced mode case compared

Two peak total contact forces were observed in the medial compartment for the unbraced mode at the opposite toe off (OTO) and the opposite initial contact (OIC) events of the gait cycle (Fig. 3a). These two peaks contribute to 88% and 79% of the total inferior forces. Compared to the unbraced mode, the 0°, 4°, and 8° brace alignment modes reduced the total contact force by 16%/46%/82% at OTO and 18%/17%/29% at OIC events, respectively (Fig. 3d). The 0°, 4°, and 8° brace alignment modes all demonstrated a significant decrease in total contact area induced on the medial tibial cartilage during the main events of the gait cycle when compared to the unbraced knee (Fig. 3b). A reduction of the total contact area by 13%/25%/58% and 2%/5%/8% was observed at OTO and OIC events, respectively, when compared to the unbraced knee (Fig. 3e). The peak contact pressures in the medial compartment during the main events of the gait cycle decreased significantly for the braced condition when compared to the unbraced condition (Figs. 3c and 4a). The 0°, 4°, and 8° brace alignment modes reduced the peak contact pressure by 4%/22%/48% at OTO and 13%/14%/27% at OIC events, respectively, compared to the unbraced knee (Fig. 3f).

### Contact mechanics in the lateral compartment

**Fig. 5:**
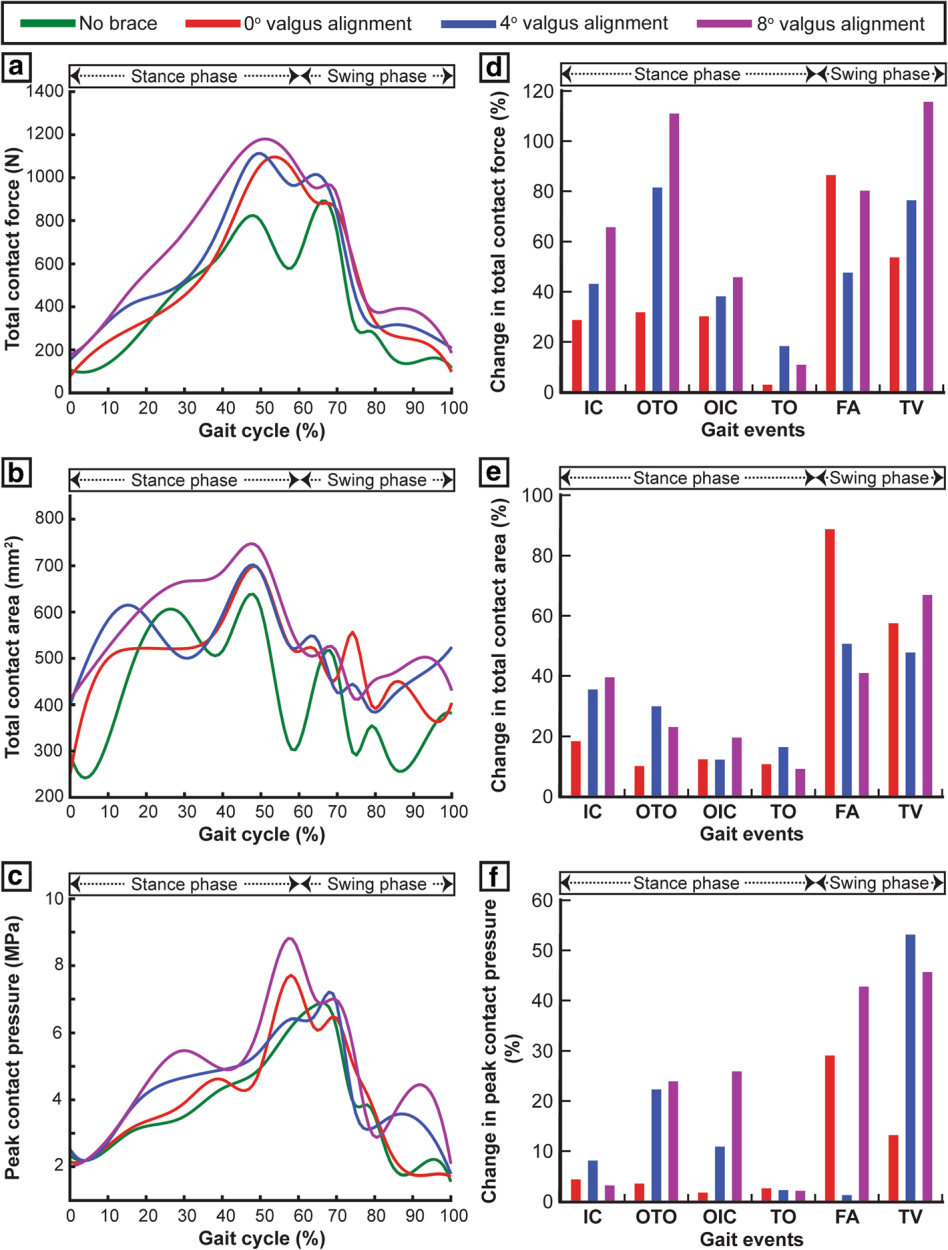
**a** Total contact force, **b** total contact area, and **c** peak contact pressure induced on the lateral tibial cartilage during one complete gait cycle for unbraced and braced modes. **d**–**f** Percentage change in the lateral compartment contact mechanics (total contact force, total contact area, and peak contact pressure) for different braced modes during the main events of the gait cycle (IC, initial contact; OTO, opposite toe off; OIC, opposite initial contact; TO, toe off; FA, feet adjacent; TV, tibia vertical) when compared with the unbraced mode

Compared to the unbraced knee, the 0°, 4°, and 8° brace alignment modes increased the total contact force by 31%/81%/110% at OTO and 30%/38%/45% at OIC events, respectively (Fig. 5a, d). These brace alignment modes all demonstrated a significant increase in total contact area induced on the lateral tibial cartilage during the critical events of the gait cycle when compared to the unbraced knee (Fig. 5b). An increase in the total contact area by 10%/30%/22% and 12%/11%/19% was observed at OTO and OIC events, respectively, when compared to the unbraced knee (Fig. 5e). The peak contact pressures in the lateral compartment during the main events of the gait cycle increased significantly when compared to the unbraced mode (Figs. 4b and 5c). The 0°, 4°, and 8° brace alignment modes increased the peak contact pressure by 4%/22%/24% at OTO and 2%/11%/26% at OIC events, respectively, compared to the unbraced knee (Fig. 5f).

### Tibial kinematics relative to the femur

**Fig. 6:**
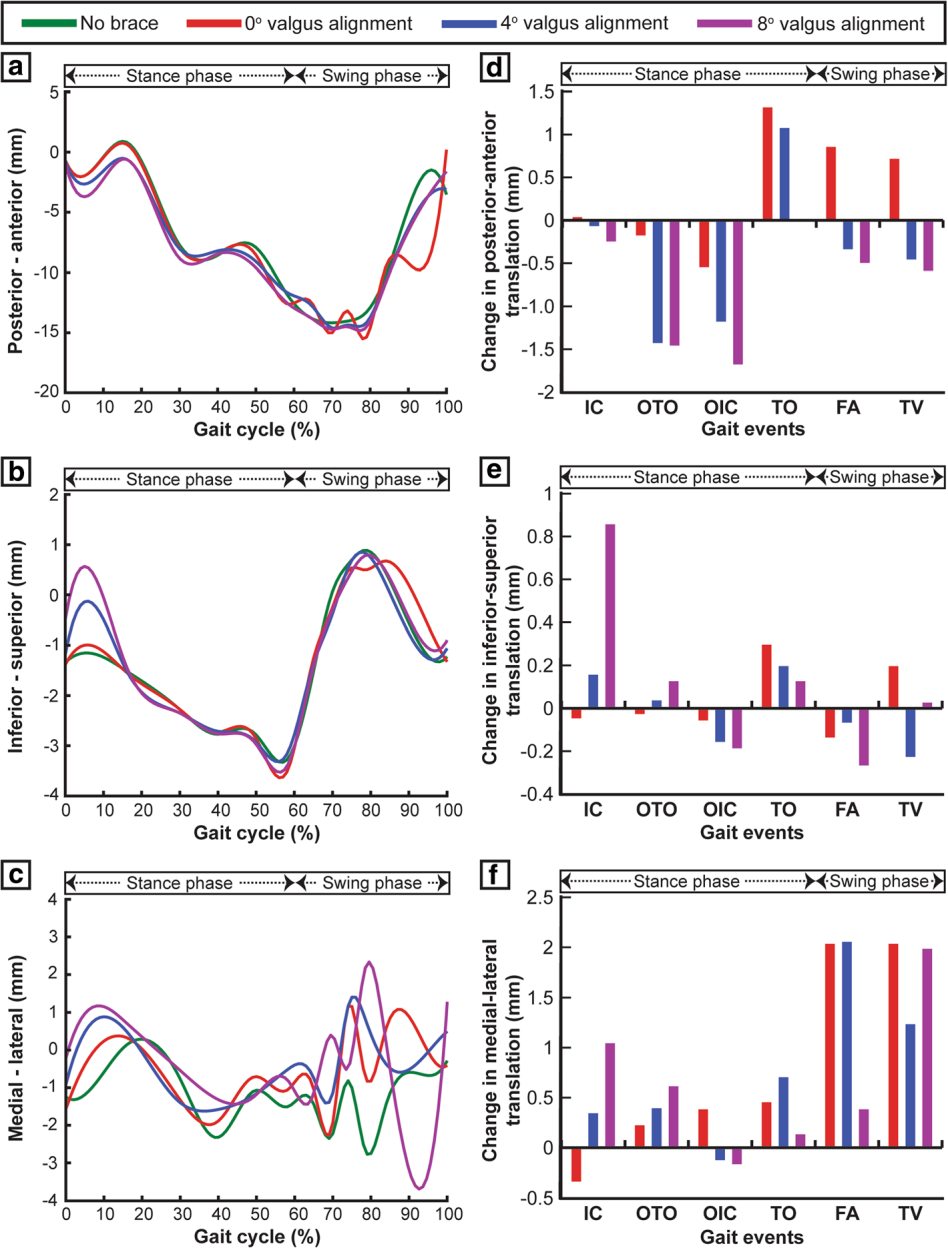
Tibial translations with respect to the femur including **a** posterior-anterior translation, **b** inferior-superior translation, and **c** medial-lateral translation induced during one complete gait cycle for unbraced and braced modes. **d**–**f** Change in tibial translations (posterior-anterior, inferior-superior, and medial-lateral) for different braced modes during the main events of the gait cycle (IC, initial contact; OTO, opposite toe off; OIC, opposite initial contact; TO,toe off; FA, feet adjacent; TV, tibia vertical) when compared with the unbraced mode

**Fig. 7:**
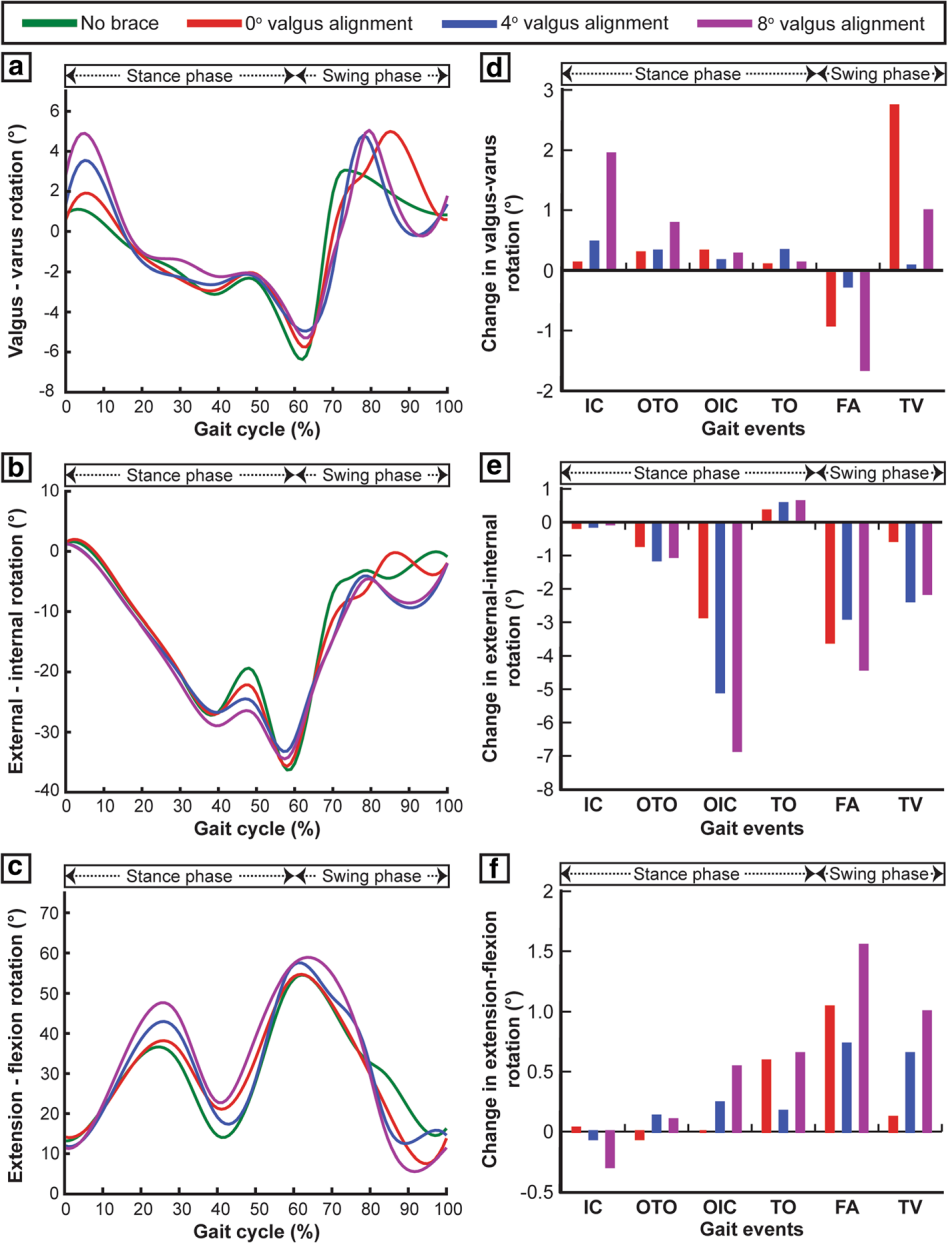
Tibial rotations with respect to the femur including **a** valgus-varus rotation, **b** external-internal rotation, and **c** extension-flexion rotation induced during one complete gait cycle for unbraced and braced modes. **d**–**f** Change in tibial rotations (valgus-varus, external-internal, and extension-flexion) for different braced modes during the main events of the gait cycle (IC, initial contact; OTO, opposite toe off; OIC, opposite initial contact; TO, toe off; FA, feet adjacent; TV, tibia vertical) when compared with the unbraced mode

The 0°, 4°, and 8° brace alignment modes all significantly increased the posterior tibial translation with respect to the femur when compared to the unbraced knee (Fig. 6a). These brace alignment modes increase the posterior translation by 0.2 mm/1.4 mm/1.5 mm at OTO and 0.5 mm/1.2 mm/1.7 mm at OIC events of the gait cycle, respectively (Fig. 6d). All brace alignment modes did not have a significant effect on the superior-inferior tibial translations (Fig. 6b, e). Compared to the unbraced mode, the 0°, 4°, and 8° brace alignment modes decreased medial tibial translations by 0.2 mm/0.4 mm/0.6 mm at OTO and 0.4 mm/0.7 mm/0.1 mm at OIC events, respectively (Fig. 6c, f). Increase in valgus brace alignment angle resulted in decreased varus-valgus and extension-flexion rotations and increased internal-external rotations during the main events of the gait cycle (Fig. 7a–f).

## Discussion

The goal of this exploratory study was to investigate the biomechanical effects of a valgus unloader brace in the arthroscopic medial meniscectomized knee joint during one complete gait cycle. While no previous studies reported the immediate effect of a valgus unloader brace on the total and peak contact forces and pressures acting in the medial compartment of the knee for the activity assessed, this study demonstrated a decrease in the total and peak contact forces and pressures in the medial compartment during one complete gait cycle. Reduction in total contact force on the medial tibial plateau by 10% has been shown to provide significant clinical advantages like reduction in pain and ameliorated joint function. Christensen et al. [34] reported a meta-analysis of randomized controlled trials that a reduction in medial load of 10% of the body weight resulted in a 28% amelioration in knee joint function. Thus, the decrease in peak contact forces and pressures in the affected compartment may translate into greater knee-related confidence.

Numerous FE studies were conducted to study the tibiofemoral contact mechanics in the medial meniscectomized knee joint [35]. However, no study has focused on determining the effectiveness of a valgus unloader brace in delaying the onset and progression of OA in the medial meniscectomized patient population. To provide confidence in the predictive ability of our FE model to estimate the contact conditions accurately, we estimated the tibial translations and rotations with respect to the femur and compared it against the values reported in the literature. The magnitude and direction of the posterior-anterior translation and the external-internal rotation of the tibia at initial contact (IC) event fall in line with the predictions reported by Andriacchi and Dyrby [36], and Lafortune et al. [37] Anterior tibial translation was observed during all gait events except IC event, and maximum anterior translation was observed at the foot adjacent (FA) event of the swing phase of the gait cycle. Many clinical studies have reported this stable orientation of the tibia in the anterior region of the knee [36–38]. The medial-lateral translations and the valgus-varus rotations of the tibia relative to the femur estimated by our FE model are in good agreement with the magnitude and the direction predicted by Lafortune et al [37] and Kozanek et al. [38] The tibial translations and rotations and the total contact force induced on the medial and the lateral compartments during the passive motion of the knee joint were estimated and compared to the experimental measurements [39, 40] to validate the FE model (refer to Additional file 1: Figure S3a–h). In conclusion, the tibial kinematics reported in our FE study is within the envelope of those reported in the literature.

We observed that a total of 56 to 89% of the inferior-superior force was transferred through the medial side of the unbraced meniscectomized knee during the stance phase of the gait cycle. This result is in good agreement with the literature that the inferior-superior force is predominantly transferred through the medial side of the meniscectomized knee [8]. The total contact force in the medial compartment is also of clinical interest, as it is related with the degeneration of patellar cartilage in the medial meniscectomized patient population while performing day-to-day activities [41]. Due to variations in the design of valgus braces, valgus alignment settings, and different measured variables, a comparison of our results with those available in the literature is limited. Pollo et al [9] used an analytical model of the knee joint to estimate the total contact force acting within the medial compartment. For 0°, 4°, and 8° brace alignment modes, they reported a reduction in the total contact force of 8%, 11%, and 17%, respectively. These findings are in consonance with the range of our data. A valgus unloader brace at 8° valgus alignment setting resulted in a maximum reduction of total contact force when compared to 4° and 0° valgus alignment angles. This result is in line with literature that the increase in valgus alignment angle will result in reduced total contact force within the medial compartment [8, 9]. This study shows that the effects of a valgus unloader brace depend on the valgus alignment angle. Since the solace and the acceptance of the patient wearing a valgus knee brace are of real significance, the amount of allowable counteracting external abduction moment is constrained. The volunteer reported discomfort when walking with the valgus brace in 8° alignment. Since the 8° valgus alignment mode would most likely not be tolerated by the medial meniscectomized patient population, a maximum reduction of the total contact force of over 47% cannot be expected aeonianly.

The peak contact pressure and the total contact area in the lateral compartment increased during the gait cycle for all braced modes when compared with the unbraced knee. This finding shows that the valgus unloader brace shifts the axial load from the medial compartment towards the lateral compartment. The lateral tibial plateau is generally thicker and has more focal thickness distribution than the medial tibial plateau [42], which makes it vulnerable to changes in contact mechanics during the gait cycle [43]. The valgus unloader brace shifts the load to regions of lateral tibial cartilage that were not accustomed to sustaining cyclic loads during gait, and this might initiate lateral cartilage degeneration in a manner akin to the medial meniscectomized knee [42, 43]. To the best of our erudition, no previous studies have reported the quantitative effect of valgus braces on the contact mechanics in the lateral compartment, which partially may expound the underutilization of valgus braces in treating the patients with medial knee OA due to the fear of damaging the healthy compartment and accelerating the degeneration process towards the stage of knee replacement.

Shifting the axial load from the affected compartment to the contralateral healthy compartment to avert or delay the onset and progression of the degenerative disease is the ultimatum of any biomechanical intervention like valgus unloader brace. Clinical benefits including reduced joint pain, increased joint range of motion, and reduced knee stiffness were reported in many previous studies after the use of a valgus unloader brace [8–10]. However, the underlying mechanisms causing these clinical benefits were never explored. In this study, the biomechanical evaluation of the contact mechanics in the medial and the lateral compartments during the gait cycle while using a valgus unloader brace provided insightful information about the conceivable underlying mechanisms responsible for the clinical benefits reported in other studies.

Strengths of our study include the evaluation of contact mechanics in the meniscectomized knee joint and the cases considered. One of the consequential advantages of this FE model is the fact that it allows a large range of motion of the knee joint. Previous FE studies have often constrained the internal-external rotations of the tibia due to modeling difficulties [35]. The relative contributions of a valgus unloader brace to the knee joint kinetics and kinematics vary based on the constraints applied by the testing equipment. Thus, the knee joint FE model with a large range of motion used in this study seems to be the most opportune for evaluating the subtle changes in knee joint mechanics during the gait cycle. Some limitations of this study warrant specifying. The FE model of the medial meniscectomized knee joint was developed using the geometric information of one subject, so care must be given while interpreting these results generally to all subjects. It is indeed probable that another medial meniscectomized knee could respond very differently to the same walking cycle. However, the main conclusions from this study will not change. Further investigation to assess the influence of age, ethnicity, body shape (hip-waist ratio, calf-thigh ratio), and BMI on the effectiveness of unloading braces is needed. Biphasic and depth-dependent material model was not used to model the articular cartilages, which might adversely affect the mechanical response of the articular cartilage [5, 35]. However, for the loading rate used in this study (approximately 0.5 Hz), the isotropic and hyperelastic non-linear neo-Hookean material model would be sufficient as the fluid will not have enough time to move inside the cartilage cells [44]. Another limitation is that the material models used for modeling the menisci and the cartilages did not include viscoelastic and swelling properties [45]. Despite these limitations, our computational model unanimously showed the biomechanical effects of valgus unloader brace in the knee joint.

## Conclusions

Our findings suggest that the use of a valgus unloader brace in the medial meniscectomized patient population decreases the mechanical load in the medial compartment by shifting them to the healthy contralateral compartment during normal day-to-day activities, like walking. Our findings further suggest that the valgus unloader brace shifts the mechanical load to the regions of lateral tibial cartilage that were not conditioned to sustain cyclic loads during gait and this might initiate lateral cartilage degeneration. This study provides a novel methodological platform to evaluate the biomechanical changes in the knee joint caused by a valgus unloader brace as well as other mechanical interventions.

## Additional file

Additional file 1: **Figure S1.** Components of quadriceps force applied to the quadriceps tendon. (a) Anterior-posterior component and (b) inferior-superior component. **Figure S2.** Gait data input for the FE model. (a) Anterior-posterior force, (b) inferior-superior force, (c) medial-lateral force, (d) valgus-varus moment, (e) external-internal moment, and (f) flexion-extension rotation. **Figure S3.** (a–f) Comparison of tibial translations and rotations induced during the passive motion of the knee joint with the cadaveric data [39], and (g–h) comparison of total contact force induced during the passive motion of the knee joint with those measured [40] during the swing phase of the gait cycle. (DOCX 1171 kb)

## Abbreviations

3D: Three dimensional
ACL: Anterior cruciate ligament
ALL: Anterolateral ligament
APL: Arcuate popliteal ligament
BMI: Body mass index
ETL: Echo train length
FA: Feet adjacent
FE: Finite element
FOV: Field of view
FSE: Fast spin echo
GRF: Ground reaction force
IC: Initial contact
LCap: Lateral capsular ligament
LCL: Lateral collateral ligament
MCap: Medial capsular ligament
MCL: Medial collateral ligament
MR: Magnetic resonance
NEX: Number of excitations
OA: Osteoarthritis
OIC: Opposite initial contact
OPL: Oblique popliteal ligament
OTO: Opposite toe off
PCL: Posterior cruciate ligament
PT: Patellar tendon
QT: Quadriceps tendon
STATEV: State variables
TE: Echo time
TO: Toe off
TR: Repetition time
TV: Tibia vertical
