# Supplementary material for: Effects of a valgus unloader brace in the medial meniscectomized knee joint: a biomechanical study
Source: J Orthop Surg Res. 2019 Feb 12;14:44. doi: 10.1186/s13018-019-1085-1 (PMC6373038; doi:10.1186/s13018-019-1085-1)
Supplement: Supplementary file 1 — Figure S1. Components of quadriceps force applied to the quadriceps tendon. (a) Anterior-posterior component and (b) inferior-superior component. Figure S2. Gait data input for the FE model. (a) Anterior-posterior force, (b) inferior-superior force, (c) medial-lateral force, (d) valgus-varus moment, (e) external-internal moment, and (f) flexion-extension rotation. Figure S3. (a–f) Comparison of tibial translations and rotations induced during the passive motion of the knee joint with the cadaveric data [39], and (g–h) comparison of total contact force induced during the passive motion of the knee joint with those measured [40] during the swing phase of the gait cycle. (DOCX 1171 kb) [file 13018_2019_1085_MOESM1_ESM.docx]

**Additional file 1**

**Effects of a Valgus Unloader Brace in the Medial Meniscectomized Knee Joint: A Biomechanical Study**

Duraisamy Shriram^1^, Go Yamako^2^, Etsuo Chosa^3^, Yee Han Dave Lee^4^, Karupppasamy Subburaj^1,5*^

^1^Engineering Product Development (EPD) Pillar, Singapore University of Technology and Design (SUTD), 8 Somapah Road, Singapore 487372

^2^Department of Mechanical Design Systems, Faculty of Engineering, University of Miyazaki, 1-1 Gakuen Kibana-dai-nishi, Miyazaki 889-2192, Japan

^3^Department of Orthopaedic Surgery, Faculty of Medicine, University of Miyazaki, 5200 Kihara, Kiyotake, Miyazaki 889-1692, Japan

^4^Department of Orthopaedic Surgery, Changi General Hospital, Singapore 529889

^5^Centre for Innovation, Changi General Hospital, Singapore 529889

*Corresponding author: Karupppasamy Subburaj, [subburaj@sutd.edu.sg](mailto:subburaj@sutd.edu.sg)

**Figure S1**

**
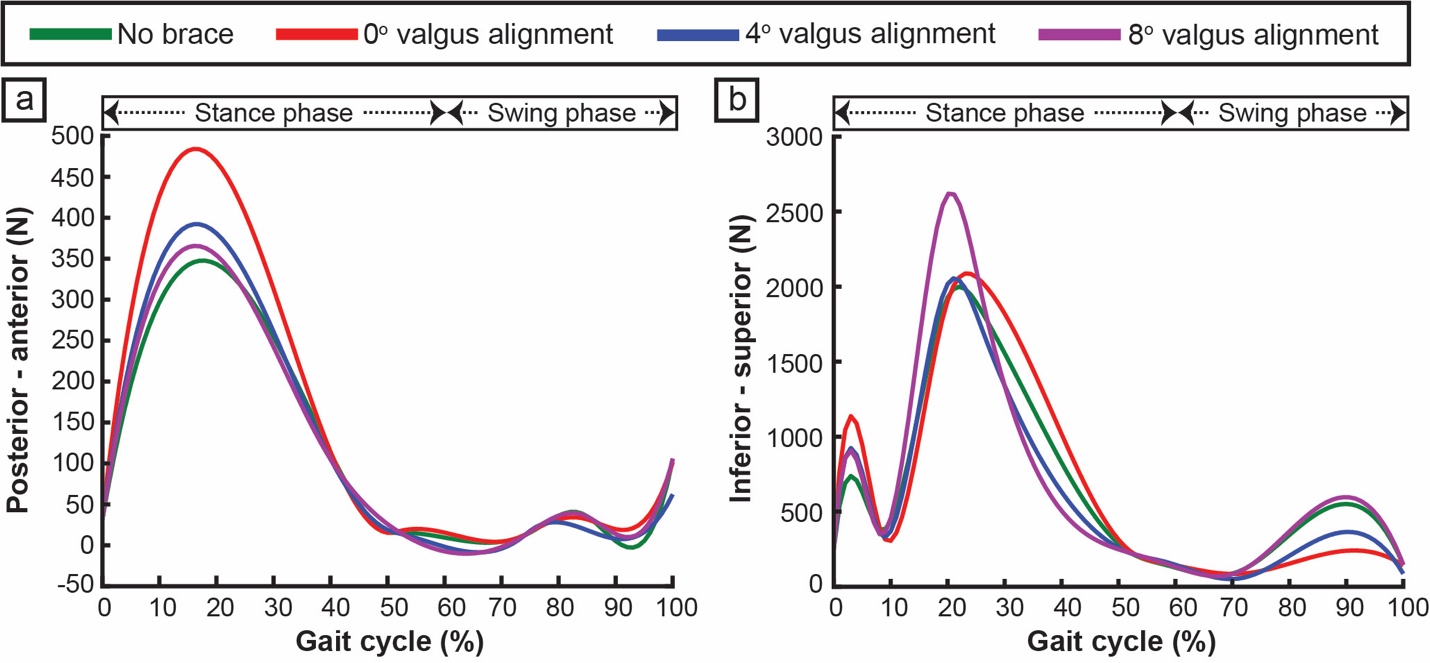
**

***Figure S1.*** *Components of quadriceps force applied to the quadriceps tendon. (a) anterior-posterior component, and (b) inferior-superior component.*

**Figure S2**

**
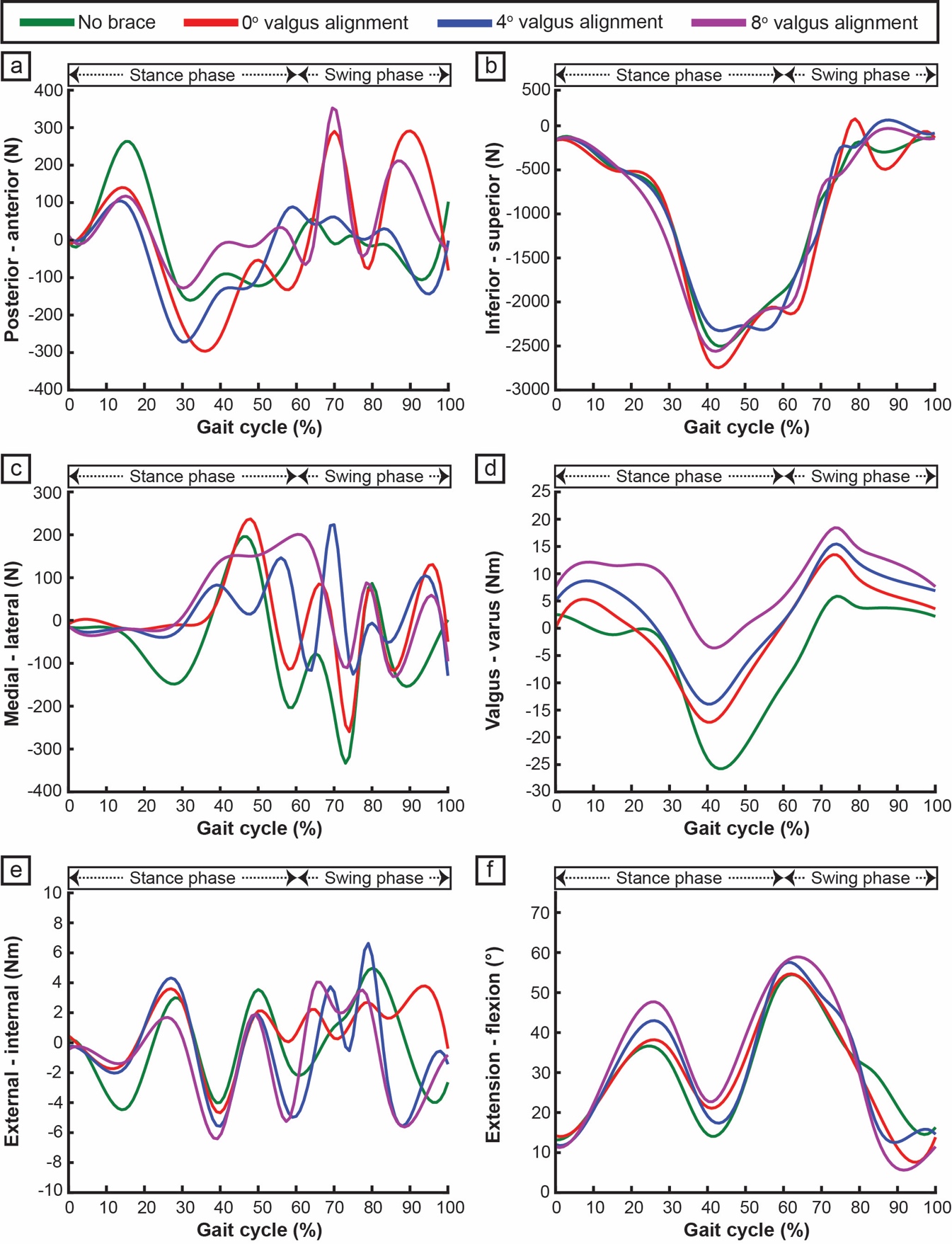
**

***Figure S2.*** *Gait data input for the FE model. (a) anterior-posterior force, (b) inferior-superior force, (c) medial-lateral force, (d) valgus-varus moment, (e) external-internal moment, and (f) flexion-extension rotation.*

**Figure S3**

**
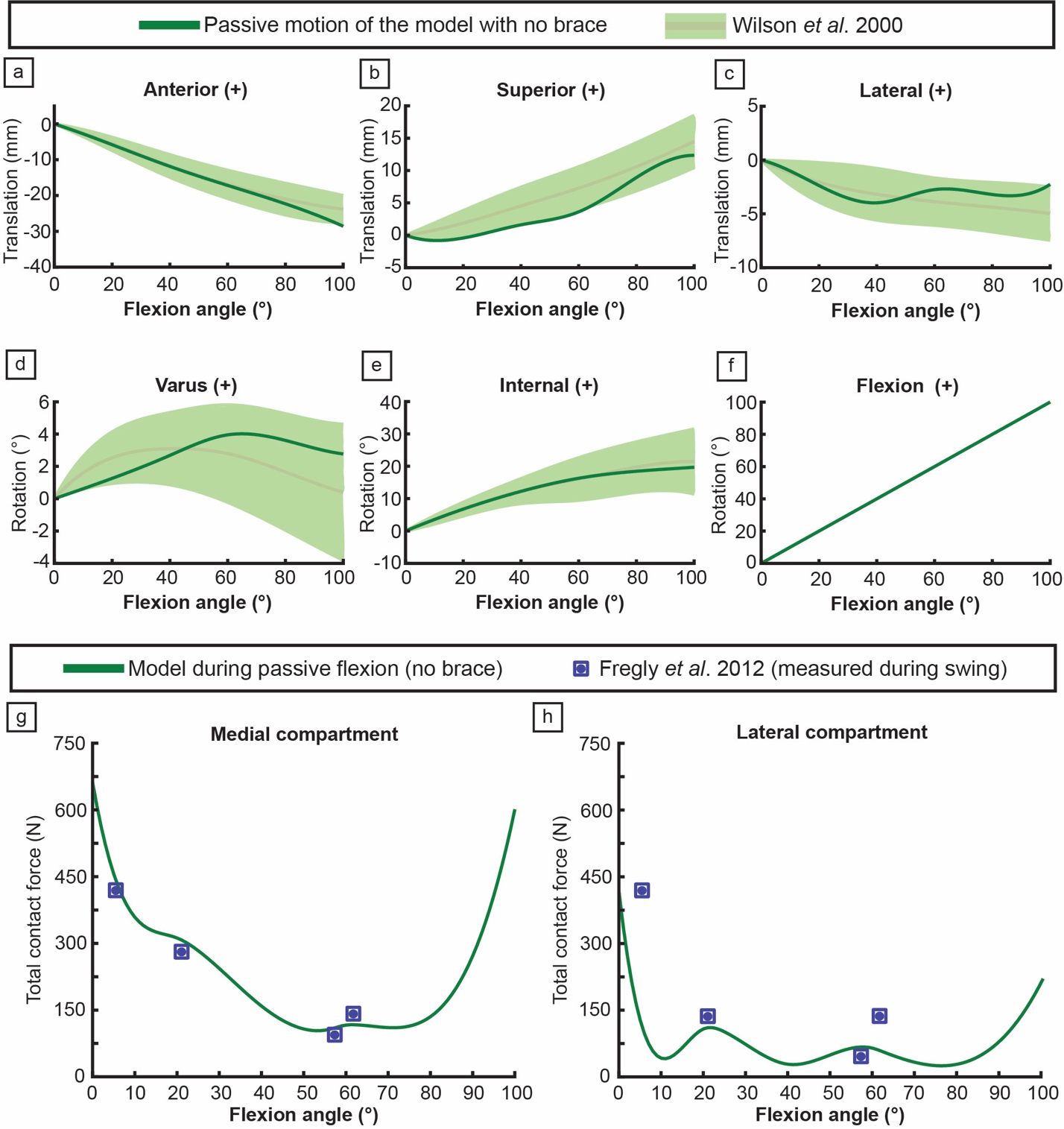
**

***Figure S3.*** *(a-f) Comparison of tibial translations and rotations induced during the passive motion of the knee joint with the cadaveric data^39^, and (g-h) comparison of total contact force induced during the passive motion of the knee joint with those measured^40^ during the swing phase of the gait cycle.*
